# Periostin Is Required for the Maintenance of Muscle Fibers during Muscle Regeneration

**DOI:** 10.3390/ijms22073627

**Published:** 2021-03-31

**Authors:** Naoki Ito, Yuko Miyagoe-Suzuki, Shin’ichi Takeda, Akira Kudo

**Affiliations:** 1 Laboratory of Molecular Life Science, Institute of Biomedical Research and Innovation (IBRI), Foundation for Biomedical Research and Innovation at Kobe (FBRI), Kobe 650-0047, Japan; naoki.ito.198571@gmail.com; 2 National Center of Neurology and Psychiatry, Department of Molecular Therapy, National Institute of Neuroscience, Tokyo 187-8502, Japan; miyagoe@ncnp.go.jp; 3 Department of Biological Information, Tokyo Institute of Technology, Yokohama 226-8501, Japan

**Keywords:** skeletal muscle regeneration, extracellular matrix, periostin

## Abstract

Skeletal muscle regeneration is a well-organized process that requires remodeling of the extracellular matrix (ECM). In this study, we revealed the protective role of periostin, a matricellular protein that binds to several ECM proteins during muscle regeneration. In intact muscle, periostin was localized at the neuromuscular junction, muscle spindle, and myotendinous junction, which are connection sites between muscle fibers and nerves or tendons. During muscle regeneration, periostin exhibited robustly increased expression and localization at the interstitial space. Periostin-*null* mice showed decreased muscle weight due to the loss of muscle fibers during repeated muscle regeneration. Cultured muscle progenitor cells from periostin-*null* mice showed no deficiencies in their proliferation, differentiation, and the expression of Pax7, MyoD, and myogenin, suggesting that the loss of muscle fibers in periostin-*null* mice was not due to the impaired function of muscle stem/progenitor cells. Periostin-*null* mice displayed a decreased number of CD31-positive blood vessels during muscle regeneration, suggesting that the decreased nutritional supply from blood vessels was the cause of muscle fiber loss in periostin-*null* mice. These results highlight the novel role of periostin in maintaining muscle mass during muscle regeneration.

## 1. Introduction

Skeletal muscle has a high regenerative ability. When skeletal muscle is damaged by external factors, inflammatory cells such as monocytes and macrophages infiltrate and phagocytose necrotic fibers [1]. Skeletal muscle regeneration is a highly organized process including activation, proliferation, and differentiation of muscle satellite cells, which are tissue-specific stem cells in skeletal muscle [2]. In parallel with the removal of necrotic fibers by inflammatory cells, satellite cells are activated to become myoblasts, which proliferate, fuse with each other or existing muscle fibers, and form a myotube, thereby regenerating myofibers. These processes are accompanied by remodeling of the extracellular matrix (ECM), which surrounds skeletal muscle fibers [3]. As the muscle grows or regenerates, ECM is repeatedly degraded and reconstructed, which enables efficient cell proliferation and migration and maintains tissue strength.

Periostin, also known as osteoblastic-specific factor 2, is specifically expressed in the osteoblast cell line MC3T3-E1 [4]. Periostin has at least 4 splice variants formed by the deletion of its C-terminal domain in mice [5]. TGF-β1 specifically induces the expression of shorter isoforms of periostin, fibronectin, and tenascin-C. Although the functional differences between the full length and the shorter isoforms of periostin are not revealed, the full length of periostin is hardly secreted, whereas the shorter isoforms are fundamentally located in the extracellular matrix to regulate cell migration and proliferation [5]. Periostin is highly expressed and localized in the ECM of the periodontal ligament and periosteum [6]. While periostin has been isolated as an osteoblast-specific molecule, subsequent studies have revealed its expression in cancer tissues, interstitial space during myocardial infarction, and skeletal muscle during muscle regeneration [7,8,9,10]. Periostin binds to ECM proteins such as collagen, fibronectin, and tenascin-C [11]. Furthermore, it maintains tissue strength by regulating collagen fiber formation through controlling cross-linked structures of type I collagen and uptake of tenascin-C into the ECM [8,12,13,14,15]. In addition, periostin binds to integrin αvβ3, αvβ5, and α6β4, and is involved in cell migration and adhesion [16,17]. Periostin also regulates angiogenesis in cancer [18]. Previous reports suggested that periostin is involved in the formation of ECM in skeletal muscle, where it is upregulated during muscle regeneration along with collagen, MMP3, MMP9, and biglycan [9,10,19]. In zebrafish, periostin is required for muscle–muscle septum connection and muscle differentiation [20]. Additionally, CD31(-) CD45(-) side population cells, which are a type of fibroblasts that highly express periostin, proliferate during muscle regeneration and promote the proliferation and migration of muscle satellite cells [21].

Lorts, A. et al. previously showed that the loss of periostin reduced fibrosis in muscular dystrophy, suggesting that periostin worsened the tissue repair process [10]. However, another previous study revealed that periostin-expressing skeletal stem cells had high regeneration capacity, and these cells were required for bone repair [22], suggesting that periostin contributed to the adequate process for tissue repair. Thus, the role of periostin during tissue repair process especially in skeletal muscle is still controversial, and the function of periostin in skeletal muscle has yet to be clarified. In this study, we aimed to clarify the function of periostin in skeletal muscle. Using periostin-null mice and immuno-histological and gene expression analyses, we found that loss of periostin caused the decreased number of muscle fibers after the induction of muscle regeneration, indicating that periostin was required to maintain muscle mass during muscle regeneration. These results highlight the novel role of periostin in maintaining muscle mass during muscle regeneration.

## 2. Results

### 2.1. Periostin Is Localized at the Neuromuscular Junction, Muscle Spindle, and Myotendinous Junction in Skeletal Muscle

**Figure 1:**
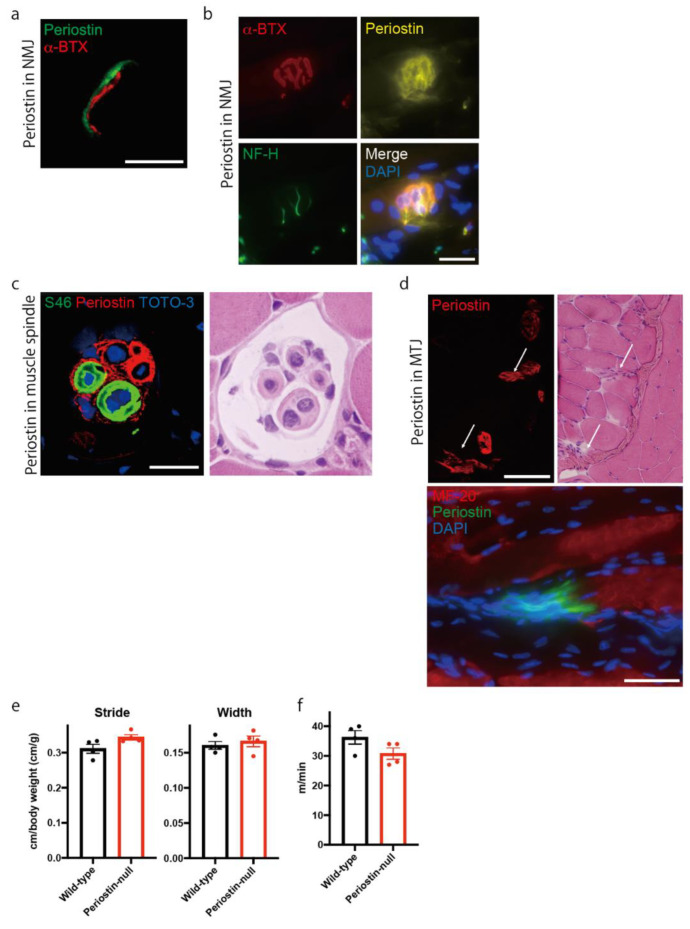
Periostin is localized at the neuromuscular junction, muscle spindle, and myotendinous junction in skeletal muscle. (**a**) Representative image of periostin localization (green) with α-BTX (red) using transverse section. Bar: 16 μm (left). (**b**) Representative image of periostin localization (yellow) with α-BTX (red) and neurofilament H (NF-H) positive-motor neuron (green) using longitudinal section (right). Bar: 25 μm. (**c**) Representative image of periostin localization (red) at the muscle spindle. Bag fibers and nuclei were stained by anti-S46 (green) and TOTO-3 (blue), respectively. Bar: 20 μm. (**d**) Upper: Representative image of periostin localization (red) at the myotendinous junction (MTJ). The serial section was stained with anti-periostin antibody (left) and H&E (right). The representative periostin-expressing MTJs were indicated by arrows. Lower: Longitudinal section was stained with anti-periostin (green) and MF-20-positive muscle fibers (red). Bar: 50 μm. (**e**) The locomotive function was analyzed by foot paint analysis. (**f**) Maximum running speed was analyzed using a treadmill. Mouse tibialis anterior muscles were used for histological analysis. *n* = 4. Statistical analysis was performed using Student’s *t*-test. Error bars indicate SEM.

Because the expression of periostin in intact muscle is not reported, we first analyzed its expression in skeletal muscle to reveal the role of periostin in skeletal muscle. Using an antibody against periostin [12], we found that periostin was expressed in the neuromuscular junction (NMJ), a connection site between skeletal muscle fiber and motor neuron (Figure 1a). Periostin expression was observed near that of α-Bungarotoxin (BTX), which binds to the acetylcholine receptor. However, periostin was not co-localized with α-BTX. To further analyze the localization of periostin in NMJ, we stained periostin with motor neuron and α-BTX using longitudinal section (Figure 1b). α-BTX-positive NMJ was surrounded by periostin, suggesting that periostin was localized at the synaptic cleft. We also observed that periostin was localized at the muscle spindle, a stretch receptor in skeletal muscle, which is innervated by the afferent nerve (Figure 1c). We used anti-S46 as a marker of bag fibers in muscle spindle. Periostin was highly expressed around S46-positive bag fibers, chain fibers, and the inner capsule. Additionally, periostin was localized at the end of muscle fibers, indicating that periostin was localized at the myotendinous junction which connects muscle fibers and tendons (Figure 1d). These results indicated that periostin was localized at the connecting sites between skeletal muscle fibers and other tissues, such as nerves or tendons, in intact muscle. Because periostin was expressed in the NMJ and muscle spindle, we hypothesized that it plays a role in locomotive function. Thus, we analyzed the walking function of periostin-null mice [12] using foot paint (Figure 1e). However, periostin-null mice showed no differences in stride length and step width. We also evaluated the maximum running speed using a treadmill. However, the maximum running speed of periostin-null mice was comparable to that of wild-type mice (Figure 1f), suggesting that locomotive and motor functions were maintained in periostin-null mice.

### 2.2. Periostin Is Expressed in the Interstitial Space during Muscle Regeneration

**Figure 2:**
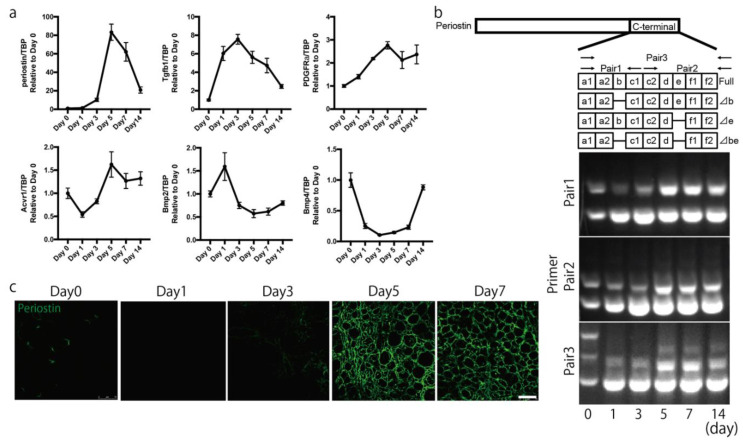
Expression of periostin is increased during muscle regeneration. (**a**) Expression of periostin, Tgfβ1, PDGFRα, Acvr1, Bmp2, and Bmp4 during muscle regeneration by quantitative PCR. (**b**) Expression of periostin isoforms during muscle regeneration. (**c**) Immunohistochemical analysis of periostin during muscle regeneration. Bar: 75 μm. *n* = 3. Error bars indicate SEM.

To analyze the role of periostin in muscle regeneration, we examined its expression pattern. We first analyzed the time course of change in periostin expression during muscle regeneration, which was induced by single injection of cardiotoxin (CTX) [23]. Following CTX injection, periostin expression gradually increased (Figure 2a), reaching a maximum on day 5 and gradually decreasing thereafter. We also analyzed the expression of potential upstream regulators of periostin. We found that the expression of TGF-β1 was increased prior to that of periostin. In addition, PDGFRα and activin receptor 1 showed expression patterns similar to that of periostin, while BMP2 and BMP4 did not, suggesting that periostin was induced by TGF-β1, PDGFRα and/or activin receptor 1 signaling during muscle regeneration. Because the expression of BMP2 and BMP4 showed the opposite expression pattern to that of periostin, periostin was unlikely to be induced by BMP2 and/or BMP4. Because periostin has several isoforms [8], we analyzed which isoforms were expressed in skeletal muscle. Periostin has four isoforms with differences in the C-terminal domain: full-length, ⊿b, ⊿e, and ⊿be. In intact muscle, both full-length and shorter isoforms were expressed (Figure 2b). The expression of the full-length isoform was diminished during muscle regeneration, after which the expression of shorter isoforms was increased. To analyze the localization of periostin during muscle regeneration, we performed immunostaining. We observed robust expression of periostin 5 and 7 days after inducing muscle regeneration (Figure 2c).

**Figure 3:**
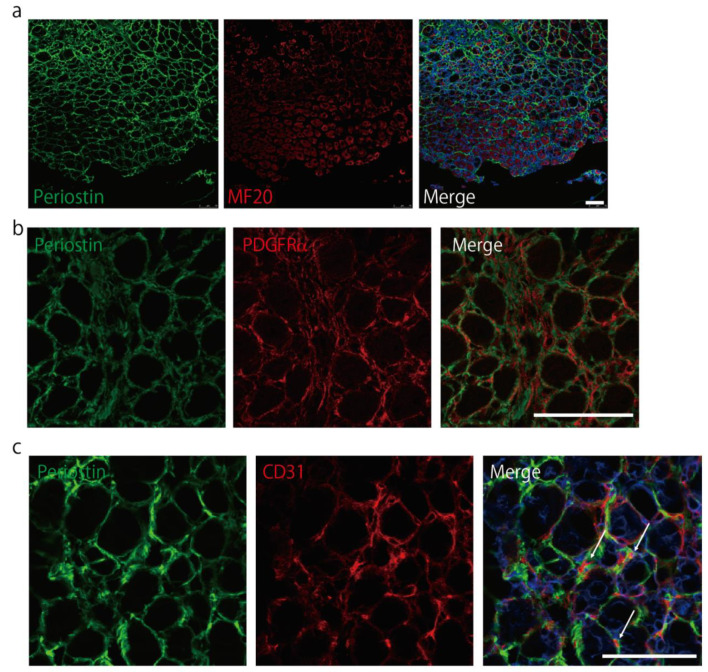
Periostin is expressed in the interstitial space during muscle regeneration. (**a**) Immunostaining of periostin (green) with MyHC (MF20, red) 5 days after the induction of muscle regeneration. Bar: 75 μm. (**b**) Immunostaining of periostin (green) and PDGFRα (red) 5 days after the induction of muscle regeneration. Bar: 75 μm. (**c**) Immunohistochemical images of periostin (green) with CD31 (red) 7 days after the induction of muscle regeneration. Arrows indicated the CD31 signals surrounded by periostin. Bar: 75 μm. *n* = 3. Error bars indicate SEM.

Periostin did not co-localize with MF20-positive myosin heavy chain (MyHC) (Figure 3a) 5 days after the induction of muscle regeneration (*p*-value of co-localization analysis was 1.0); however, similar to PDGFRα, periostin was expressed in the interstitial space during muscle regeneration (Figure 3b). We also found that some CD31 signals were surrounded by periostin 7 days after inducing muscle regeneration (Figure 3c), suggesting supportive effects of periostin on CD31. These results indicated that periostin was expressed in the interstitial space during muscle regeneration.

### 2.3. Loss of Periostin Caused a Decrease in Muscle Fibers during Repeated Muscle Regeneration

**Figure 4:**
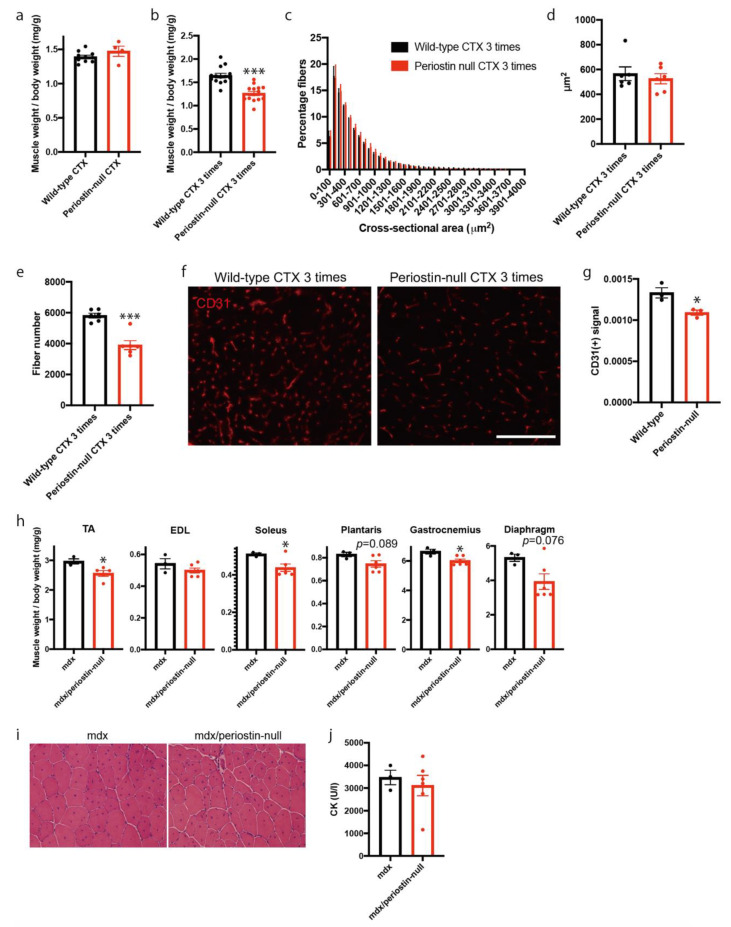
Loss of periostin causes a decrease in muscle fiber number during repeated muscle regeneration. (**a**) Muscle weight 7 days after the induction of muscle regeneration. *n* = 4–10. (**b**) Muscle weight 7 days after repeated induction of muscle regeneration. *n* = 14. (**c**) CSA of regenerating muscle fiber after repeated muscle regeneration. *n* = 6. (**d**) The average of CSA. *n* = 6. (**e**) The number of muscle fibers after repeated muscle regeneration. *n* = 6. (**f**) Immunostaining of CD31 after repeated muscle regeneration. Bar: 75 μm. (**g**) The number of CD31-positive blood vessels. *n* = 3. (**h**) Muscle weight of 12-week-old mdx and mdx/periostin-null mice. *n* = 3–6. (**i**) Representative H&E staining of mdx and mdx/periostin-null mice. (**j**) Plasma CK levels in mdx and mdx/periostin-null mice. *n* = 3–6. * *p* < 0.05 and *** *p* < 0.001 by Student’s *t*-test. Error bars indicate SEM.

To analyze the function of periostin in skeletal muscle regeneration, we injected CTX into the tibialis anterior (TA) muscles of periostin-null mice. We first analyzed the weights of TA muscles 7 days following CTX injection (Figure 4a). We observed a statistically significant decrease in muscle weight in CTX-injected wild-type mice compared with that in untreated mice (data not shown). However, this decreased weight was comparable to that of periostin-null mice (Figure 4a). Therefore, we repeatedly induced muscle regeneration by injecting CTX once per week. Muscle weights were measured three times after CTX injection. We found that muscle weight decreased by approximately 30% in periostin-null mice compared to that of wild-type mice (Figure 4b). To elucidate the reason for this decrease, we analyzed the cross-sectional area (CSA) of muscle fibers and counted the number of muscle fibers. The CSA in periostin-null mice was comparable to that in wild-type mice (Figure 4c,d). However, the total number of skeletal muscle fibers was significantly decreased in periostin-null mice (Figure 4e), indicating that the loss of muscle fibers led to decreased muscle weight in periostin-null mice. Because periostin was expressed in the interstitial space and some CD31 signals were surrounded by periostin during muscle regeneration (Figure 3c), we counted the number of CD31-positive blood vessels after repeatedly inducing muscle regeneration. We found that CD31-positive blood vessels were significantly decreased in periostin-null mice (Figure 4f,g). These results suggested that loss of periostin caused a decrease in muscle fiber number during repeated muscle regeneration, possibly due to the diminished nutritional supply from blood vessels.

Based on the above observations, we hypothesized that periostin has a protective role in muscular dystrophy which is characterized by the progressive degeneration of muscle fibers. Thus, we crossed periostin-null mice with mdx mice, a mouse model of Duchenne muscular dystrophy [24], and analyzed the muscle weight of offspring. We observed significant decreases in the weights of the TA, soleus, and gastrocnemius of 12-week-old mdx/periostin-null mice (Figure 4h). Other skeletal muscles, including the plantaris and diaphragm, also tended to decrease in muscle weight. However, mdx/periostin-null mice exhibited no apparent differences in histology (Figure 4i) and had comparable plasma creatine kinase levels to those of control mice (Figure 4j).

### 2.4. Loss of Periostin in Mice with a DBA/2 Genetic Background Delayed Muscle Regeneration

**Figure 5:**
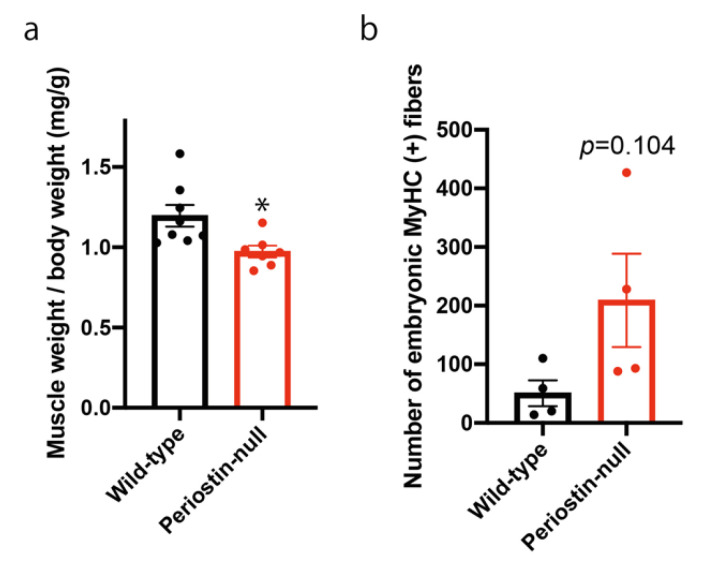
Loss of periostin delayed muscle regeneration in mice with a DBA/2 background. (**a**) Muscle weight 7 days after the induction of muscle regeneration. *n* = 7–8. (**b**) Number of embryonic MyHC-positive fibers 14 days after the induction of muscle regeneration. *n* = 4. * *p* < 0.05 by Student’s *t*-test. Error bars indicate SEM.

Because regeneration ability is affected by genetic background [25], we generated DBA/2-periostin-null mice, and induced muscle regeneration. Seven days after a single injection of CTX, we observed a decrease in muscle weight in DBA/2-periostin-null mice (Figure 5a). Although it was not statistically significant, DBA/2-periostin-null mice exhibited increased embryonic MyHC (Figure 5b), suggesting that DBA/2-periostin-null mice had delayed muscle regeneration.

### 2.5. The Function of Satellite Cells was Not Impaired in Periostin-Null Mice

**Figure 6:**
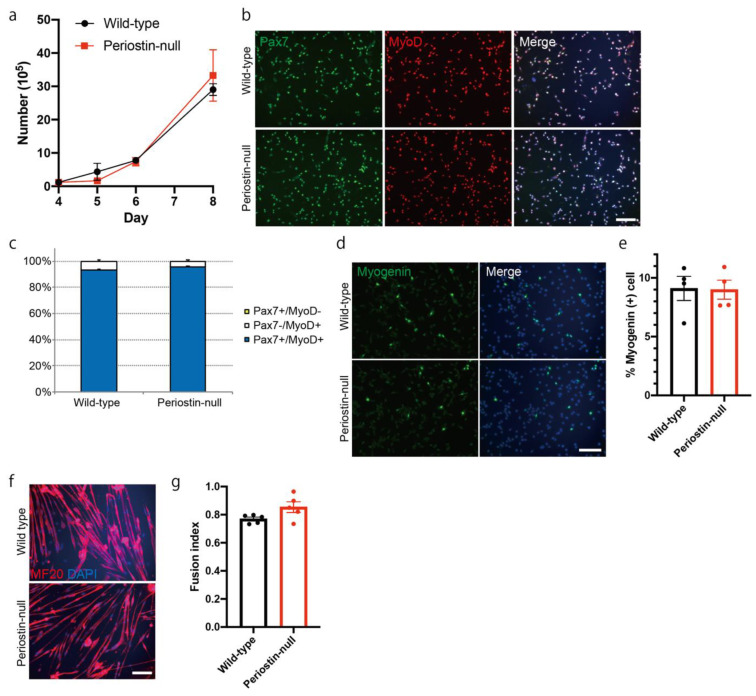
Proliferation and differentiation of muscle progenitor cells are not impaired in periostin-null mice. (**a**) Time-course changes in the number of cultured muscle progenitor cells. *n* = 4. (**b**) Immunocytochemical analysis of Pax7 and MyoD in muscle progenitor cells from periostin-null mice. Bar: 200 μm. (**c**) Ratio of Pax7+/MyoD-, Pax7-/MyoD+, and Pax7+/MyoD+ cells. *n* = 4. (**d**) Immunocytochemical analysis of myogenin in muscle progenitor cells from periostin-null mice. Bar: 200 μm. (**e**) Quantitative analysis of myogenin-positive cells. *n* = 4. (**f**) Immunocytochemical analysis of MF-20 in differentiated muscle progenitor cells from periostin-null mice. Bar: 200 μm. (**g**) Quantitative analysis for the fusion index. *n* = 5. Error bars indicate SEM.

One possible cause of muscle fiber loss (Figure 4) and delayed muscle regeneration (Figure 5) in periostin-null mice is the decreased function of muscle stem/progenitor cells. Thus, we analyzed the proliferation and differentiation of muscle progenitor cells by culturing satellite cells from periostin-null mice. Primary muscle satellite cells were prepared by isolating single muscle fibers. Upon counting the total cell number, we found that the proliferation of muscle progenitor cells was not impaired in periostin-null mice (Figure 6a). We also analyzed the expression of Pax7 and MyoD, which are key transcription factors that regulate stemness and activation of muscle stem/progenitor cells [2] (Figure 6b,c). Pax7 and MyoD expression and the ratios of Pax7+/MyoD-, Pax7-/MyoD+, and Pax7+/MyoD+ cells in muscle progenitor cells from periostin-null mice were comparable to those from wild-type mice. To examine muscle progenitor cell differentiation, we analyzed the expression of myogenin, a myogenic transcriptional factor that induces differentiation [2]. However, there was no difference in the number of myogenin-positive cells in periostin-null mice (Figure 6d,e). We induced the differentiation of muscle progenitor cells by replacing the growth medium with differentiation medium and then analyzed the fusion index by counting the number of MyHC-positive, fused differentiated cells (Figure 6f,g). However, there were no differences in the fusion index in periostin-null mice. These results indicated that the proliferation and differentiation of muscle progenitor cells from periostin-null mice in vitro was not impaired, suggesting that the decreased muscle weight in periostin-null mice was not due to the impaired function of muscle stem/progenitor cells.

## 3. Discussion

In this study, we showed the novel role of periostin during muscle regeneration. We found that loss of periostin caused the decreased number of muscle fibers after the induction of muscle regeneration, indicating that periostin was required to maintain muscle mass during muscle regeneration. 

In intact muscle, periostin was localized at the NMJ, muscle spindle, and myotendinous junction (Figure 1), which are connection sites between skeletal muscle fibers and nerves or tendons. By expressing specific proteins, including dystrophin, dystrophin-glycoprotein complex, α7-integrin, and tenascin-C, these areas had stronger and more specific ECM proteins than other areas in skeletal muscle [26,27,28], suggesting that periostin had some roles in maintaining the strength of ECM in specific connection sites in skeletal muscle.

After the induction of muscle regeneration, periostin expression was increased in the interstitial space (Figure 2 and Figure 3). Although C57BL/6-periostin-*null* mice displayed no abnormalities after a single CTX injection, they showed decreased muscle weight and loss of muscle fibers after repeated muscle regeneration (Figure 4). A similar phenotype was also observed in *mdx*-periostin *null* mice. Furthermore, DBA/2-periostin-*null* mice showed a more severe phenotype accompanied by remaining embryonic MyHC (Figure 5). The expression of ECM proteins, such as periostin, collagen, tenascin-C, and fibronectin, increases during muscle regeneration [9]. Since periostin binds to collagen, fibronectin, and tenascin-C, the interaction of periostin with other ECM proteins might be involved in the recovery process of muscle tissue. Our results suggested that periostin was induced by TGF-β1 and/or PDGFRα (Figure 2a). Regarding PDGFRα, a recent observation indicated that PDGF-BB, a ligand of PDGFRα, induced periostin transcription [29]. Thus, upregulation of PDGFRα possibly enhanced periostin expression to construct the ECM structure for muscle regeneration. Furthermore, considering that periostin-*null* mice showed a decrease in CD31-positive blood vessels (Figure 4f,g), and some CD31 signals were surrounded by periostin during muscle regeneration (Figure 3c), periostin might regulate angiogenesis during muscle regeneration. The role of periostin in angiogenesis was also reported in several periostin-expressing tumor cells [30,31]. In skeletal muscle, CD31(-) CD45(-) side population cells, which are a type of mesenchymal fibroblasts that proliferate during muscle regeneration, highly expressed several ECM proteins, including periostin, according to microarray analysis [21]; this suggested that periostin was involved in the angiogenic function in periostin-expressing mesenchymal fibroblasts during muscle regeneration. The ECM contributes to growth factor release and the activation and migration of muscle satellite cells during muscle regeneration [3,21,32,33,34]. However, because the proliferation and differentiation of muscle progenitor cells from periostin-*null* mice were not impaired in vitro (Figure 6), the impaired muscle regeneration was not likely due to the decreased function of muscle stem/progenitor cells. Although our results suggested that a decrease in muscle fiber number in periostin-null mice was possibly due to the diminished nutritional supply from blood vessels, our results did not deny other possibilities, because periostin was involved in several signaling pathways and the cross-talk between muscle fibers and ECM.

Analysis of periostin isoforms revealed that the expression patterns of each isoform differed between intact and regenerating muscle (Figure 2b); additionally, these patterns showed similar changes in cardiac muscle during acute myocardial infarction [8]. Interestingly, the expression of the full-length isoform was diminished in regenerating muscle, suggesting that it was largely localized at the intact NMJ, muscle spindle, or myotendinous junction. Considering the differences in periostin localization between intact and regenerating muscle (Figure 1 and Figure 2), the full-length isoform and shorter isoforms might have different functions in skeletal muscle.

Previously, Lorts, A. et al. reported that periostin expression was increased in muscular dystrophy, and the loss of periostin led to the partial rescue of dystrophic phenotypes, such as fibrosis [10], which was opposite to our current findings. Hara, M. et al. also reported that the loss of periostin during laceration-induced muscle injury resulted in reduced fibrosis [19]. These differences were possibly explained by differences in the experimental models. Here, we used a single or repeated muscle regeneration model using CTX and *mdx* mice, a mouse model of Duchenne muscular dystrophy caused by a mutation in the dystrophin gene [24]. On the contrary, Lorts, A. et al. used a cold injury model and δ-sarcoglycan-*null* mice, a mouse model of Limb-Girdle muscular dystrophy caused by impaired sarcoglycan genes, which encode the key components of the dystrophin–glycoprotein complex [35]. Previously, Turk, R. et al. performed omics analysis to compare *mdx* and δ-sarcoglycan-*null* mice and reported some differences in specific protein clusters related to inflammation and cell adhesion [36]. Although precise mechanisms must be clarified in the future, these differences in molecular pathophysiology might affect the function of periostin in muscle regeneration.

Taken together, our data indicate the protective role of periostin in muscle regeneration. Our findings might contribute to the development of new therapeutics for muscular degenerative diseases.

## 4. Materials and Methods

### 4.1. Animals

C57BL/6 and DBA/2 mice were purchased from Nihon CREA (Tokyo, Japan). C57BL/6-*mdx* mice were a kind gift from Dr. Toshikuni Sasaoka (National Institute for Basic Biology, Aichi, Japan). The generation and characteristics of periostin-*null* mice have been described elsewhere [12]. To generate DBA/2-periostin-*null* mice, C57BL/6-periostin-*null* mice were crossed with DBA/2 mice at least four times. All mice were housed at the institutional animal facility (NCNP, Tokyo, Japan). All animal procedures were approved by the Experimental Animal Care and Use Committee at the NCNP (Approval number: 2016001, Approval date: 2/25/2016). All experimental methods were performed in accordance with this approved guideline.

### 4.2. Exercise Model

The treadmill exercise model was performed as described previously with minor modification [37]. Briefly, the mice were placed on MK-680S treadmill (Muromachi Kikai, Tokyo, Japan). The mice were forced to run at 5 m/min for the first 5 min. Then, the speed was increased by 1 m/min until the mice could not run. The maximum running speed was assessed.

### 4.3. Materials

Antibodies are summarized in Table 1. The generation and characteristics of the anti-periostin antibody have been described elsewhere [12]. Alexa-conjugated α-BTX (B13423) was purchased from Thermo Fisher Scientific (Waltham, MA, USA). Anti-S46 was used for a marker for bag fibers in muscle spindle. 

### 4.4. RNA Isolation and Reverse Transcription–Polymerase Chain Reaction Analysis (RT-PCR)

RNA isolation and subsequent PCR analysis were performed as described previously, with minor modifications [38]. Briefly, TRIzol (Invitrogen, Carlsbad, CA, USA) was used for the isolation of total RNA. QuantiTect Reverse Transcription Kit (Qiagen, Hilden Germany) was used for the synthesis of single-strand cDNA. ExTaq (Takara, Tokyo, Japan) was used for conventional PCR. For quantitative RT-PCR, SYBR Premix Ex Taq II (Takara) on a MyiQ single-color system (Bio-Rad, Hercules, CA, USA) was used for the evaluation of the expression level of each gene. The primer sequences for RT-PCR are summarized in Table 2. For quantitative RT-PCR, the expression level of each gene was normalized to that of TATA-binding protein (TBP) or 18s rRNA.

### 4.5. Histological and Immunohistochemical Analysis, and Immunocytochemistry

Histological and immunohistochemical analysis were performed as described previously, with minor modifications [37]. Briefly, for transverse section, TA muscles were cut into 8 μm cross-sections by cryostat. After air-drying, the slides were stained with hematoxylin & eosin (H&E), and photographed using a DP71 digital camera system (Olympus, Tokyo, Japan). For longitudinal section, TA muscles were fixed with 4% paraformaldehyde/phosphate-buffered saline (PBS) for 30 min, then transferred to 10% sucrose/PBS and 20% sucrose/PBS for cryoprotection. TA muscles were cut into 15 μm cross sections by cryostat. For immunohistochemical analysis, the slides were fixed with cooled acetone for 10 min for transverse section. For longitudinal section, the slides were incubated with 0.1% triton-X100/PBS for 10 min for permeabilization without fix. The air-dried sections were blocked with 5% goat serum in 1% bovine serum albumin (BSA)/PBS for 15 min, and then incubated with anti-periostin, anti-S46, anti-BF-45, α-BTX (1:1000), anti-CD31, anti-PDGFRα, anti-MF20, anti-laminin-α2, or anti-neurofilament H in 1% BSA/PBS at 4 °C overnight. The sections were washed with PBS and incubated with Alexa Fluor 488- or Alexa Fluor 594-labeled secondary antibodies (1:1000, Thermo Fisher Scientific) in 1% BSA/PBS. Nuclei were stained with DAPI (Vector, San Francisco, CA, USA) after several washings with PBS. Immunofluorescence-stained images were evaluated by fluorescence microscope BZ-9000 (Keyence, Osaka, Japan) or Leica confocal microscope (TCS-SP5). All muscle fibers in TA muscle were counted and analyzed for CSA analysis. In addition, all CD31-positive signals in TA muscle were counted. CSAs and CD31-positive signals were determined using a Dynamic Cell Count software in BZ-9000. CD31-positive signals were normalized by the analyzed area. Co-localization analysis was performed by ImageJ.

Immunocytochemistry was performed as described previously, with minor modifications [39]. Briefly, cells were fixed with 4% paraformaldehyde/PBS for 10 min at room temperature. After several washings with PBS, fixed cells were treated with 0.1% Triton X-100/PBS for 10 min and blocked with 5% goat serum in 2% BSA/PBS for 15 min. Cells were then incubated with anti-Pax7, anti-MyoD, anti-myogenin, or anti-MF20 antibody in 2% BSA/PBS at 4 °C overnight. After several washings with PBS, the slides were incubated with Alexa Fluor 488- or Alexa Fluor 594-labeled secondary antibodies (1:1000; Thermo Fisher Scientific) in 2% BSA/PBS. Nuclei were stained with DAPI (Vector, San Francisco, CA, USA) after several washings with PBS. Images of immunofluorescent staining were evaluated by fluorescence microscopy (Olympus).

### 4.6. Single Muscle Fiber Preparation and Cell Culture

Single muscle fiber preparation and primary cell culture were performed as described previously, with minor modifications [39]. Briefly, extensor digitorum longus (EDL) muscles were isolated from 12–16-week-old mice. These muscles were dissociated with type 1 collagenase (Worthington, Lakewood, NJ, USA). Isolated EDL muscles were incubated in 0.2% type 1 collagenase/Dulbecco’s Modified Eagle Medium (DMEM) for 80–90 min. By gentle pipetting, dissociated muscles were unraveled. Isolated muscle fibers were plated on Matrigel (Matrigel-Growth Factor Reduced, BD Biosciences)-coated culture dishes. Isolated muscle fibers with primary satellite cells were cultured in DMEM (high glucose, sodium pyruvate, and GlutaMAX supplement; Thermo Fisher Scientific) supplemented with 20% fetal bovine serum, 1% chick embryo extract (US Biological, Salem, MA, USA), and 1% penicillin-streptomycin (Thermo Fisher Scientific) at 37 °C with 5% CO_2_. All plastic dishes were coated with Matrigel. The medium was changed every 2 days. Differentiation to myotubes was induced by replacing medium with DMEM supplemented with 2% horse serum and 1% penicillin-streptomycin.

### 4.7. Induction of Muscle Regeneration

To induce skeletal muscle regeneration, 100 μL of 10 μM CTX (Sigma-Aldrich, St. Louis, MO, USA) were injected into the TA/EDL muscles, as described previously [39].

### 4.8. Statistical Analysis

All values are expressed as mean ± standard error of the mean (SEM). The significance of differences was assessed by Student’s *t*-test using Prism 8. Probabilities less than 5% (*, *p* < 0.05) or 0.1% (***, *p* < 0.001) were considered to be statistically significant.

## Figures and Tables

**Table 1:** Summary of antibodies.

Antibody	Clone	Dilution	Source, Catalog Number
rabbit anti-periostin	polyclonal	300	Reference [12]
mouse anti-Neurofilament H (NF-H), Nonphosphorylated	SMI32	1500	Bioletend (San Diego, CA, USA), 801701
mouse anti-S46	S46	800	Developmental Studies Hybridoma Bank (Iowa City, IA, USA), S46
mouse anti-BF-45	BF-45	40	Developmental Studies Hybridoma Bank (Iowa City, IA, USA), BF-45
rat-anti-CD31	390	100	Immunotech (Ocala, FL, USA)
rat-anti-PDGFRa	APA5	2000	BD Pharmingen (Franklin Lakes, NJ, USA), 558774
mouse- anti-MF20	MF20	500	R&D Systems (Minneapolis, MN, USA), MAB4470
rat anti-laminin-a2	4H8-2	200	Alexis Biochemicals (San Diego, CA, USA), ALX-804-190
mouse anti-Pax7	PAX7	80	Santa Cruz Biotech (Santa Cruz, CA, USA), sc-81648
mouse anti-MyoD	5.8A	400	Santa Cruz Biotech (Santa Cruz, CA, USA), sc-32758
mouse anti-myogenin	F5D	1000	Santa Cruz Biotech (Santa Cruz, CA, USA), sc-12732

**Table 2:** Summary of primer sequence.

Primer Sequence	
periostin for all isoforms	5′-atgtacaacaatctggggcttt-3′
	5′-cgacaccatttgtggcaatc-3′
PDGFRa	5′-gcatcttcgacaacctctacac-3′
	5′-accatcatgccaggataggg-3′
TGFb1	5′-cagagaagaactgctgtgtgcg-3′
	5′-cgggttgtgttggttgtagagg-3′
periostin pair 1	5′-gataaatacatccaaatcaagtttgttcg-3′
	5′-cgtggatcacttctgtcaccgtttcgc-3′
periostin pair 2	5′-ctgaaaaacagactcgggaagaacg-3’
	5′-aaactctgtggtctggcctctggg-3′
periostin pair 3	5′-gataaaatacatccaaatcaagtttgttcg-3′
	5′-ctgaaaaacagactcgggaagaacg-3′
BMP2	5′-cagtgggagagcttcgacgtca-3′
	5′-ggagacacctgggttctcctct-3′
BMP4	5′-ggccaaacgtagtcccaagca-3′
	5′-ttccagcccacgtcactgaag-3′
Acvr1	5′-aggagagtcaatgctgtcct-3′
	5′-gagacatctgcttccgtcaa-3′
18s rRNA	5′-taccctggcggtgggattaac-3′
	5′-cgagagaagaccacgccaac-3′
TATA-binding protein (TBP)	5′-cagcctcagtacagcaatcaac-3′
	5′-taggggtcataggagtcattgg-3′

## Data Availability

The data presented in this study are contained within this article.
